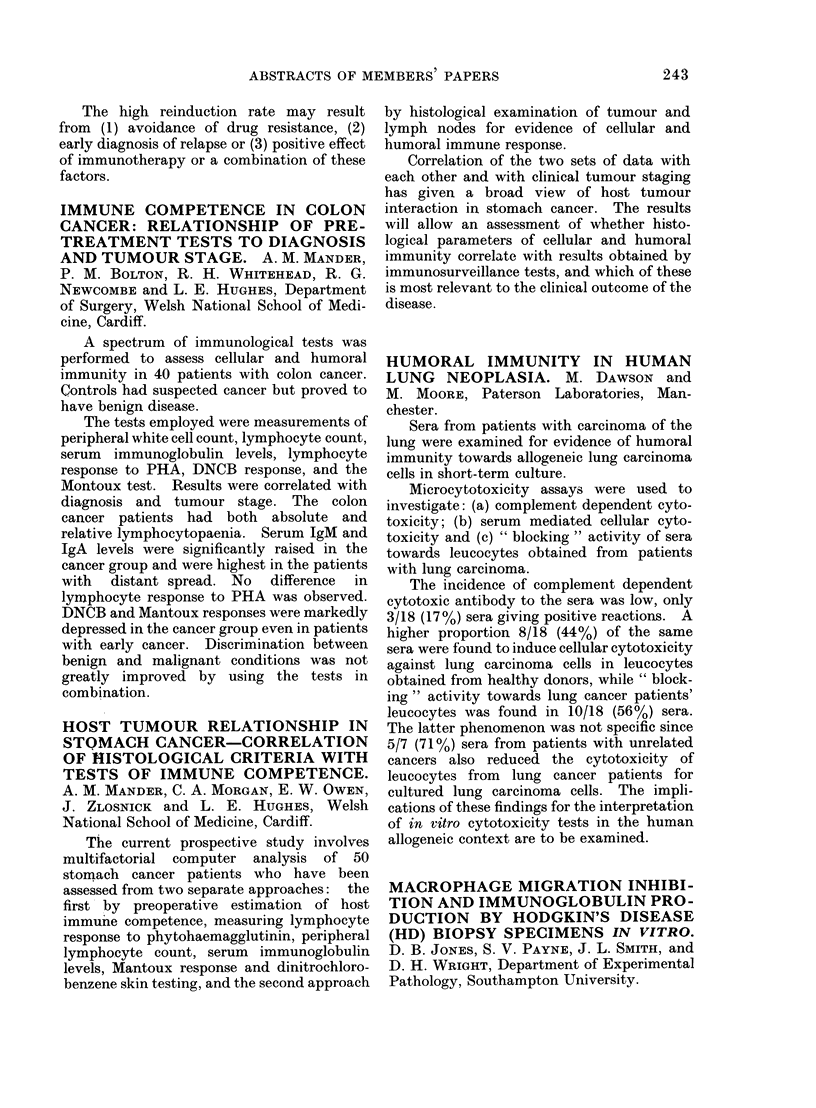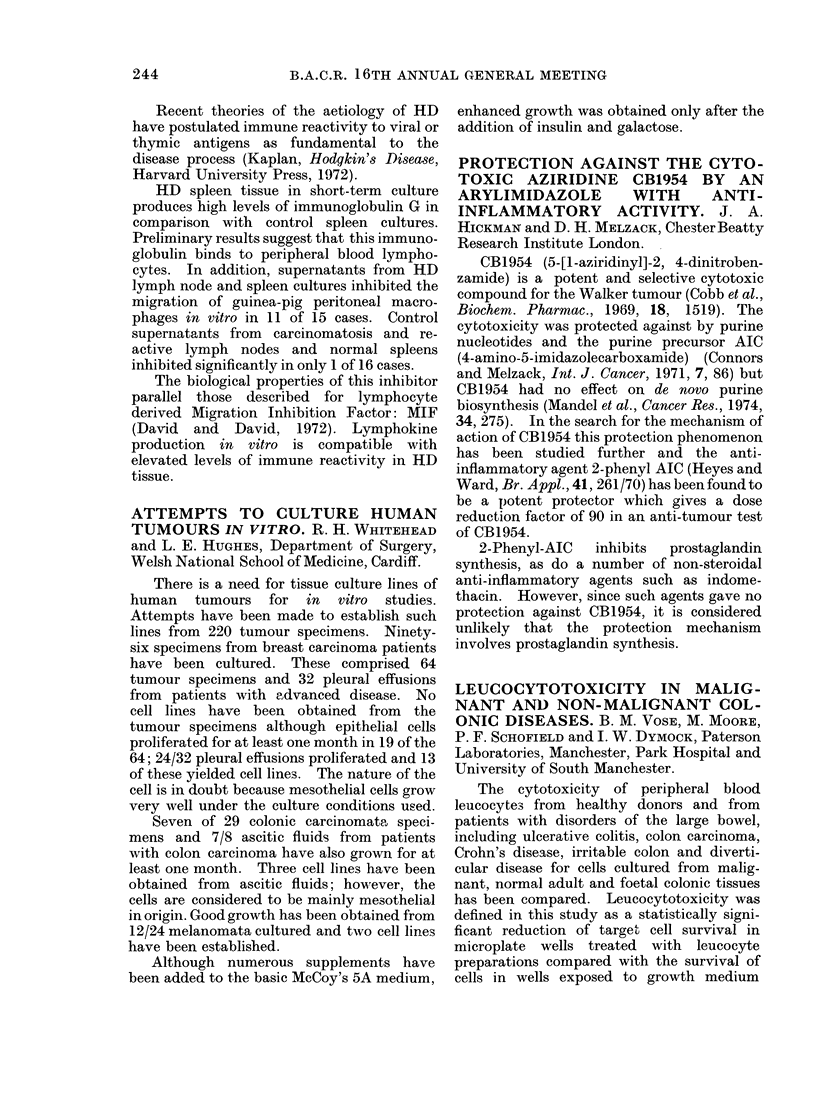# Proceedings: Macrophage migration inhibition and immunoglobulin production by Hodgkin's disease (HD) biopsy specimens in vitro.

**DOI:** 10.1038/bjc.1975.167

**Published:** 1975-08

**Authors:** D. B. Jones, S. V. Payne, J. L. Smith, D. H. Wright


**MACROPHAGE MIGRATION INHIBITION AND IMMUNOGLOBULIN PRODUCTION BY HODGKIN'S DISEASE (HD) BIOPSY SPECIMENS *IN VITRO*.** D. B. JONES, S. V. PAYNE, J. L. SMITH, and D. H. WRIGHT, Department of Experimental Pathology, Southampton University.

Recent theories of the aetiology of HD have postulated immune reactivity to viral or thymic antigens as fundamental to the disease process (Kaplan, *Hodgkin's Disease*, Harvard University Press, 1972).

HD spleen tissue in short-term culture produces high levels of immunoglobulin G in comparison with control spleen cultures. Preliminary results suggest that this immunoglobulin binds to peripheral blood lymphocytes. In addition, supernatants from HD lymph node and spleen cultures inhibited the migration of guinea-pig peritoneal macrophages *in vitro* in 11 of 15 cases. Control supernatants from carcinomatosis and reactive lymph nodes and normal spleens inhibited significantly in only 1 of 16 cases.

The biological properties of this inhibitor parallel those described for lymphocyte derived Migration Inhibition Factor: MIF (David and David, 1972). Lymphokine production *in vitro* is compatible with elevated levels of immune reactivity in HD tissue.